# Calculating the Costs of Implementing Integrated Packages of Community Health Services: Methods, Experiences, and Results From 6 sub-Saharan African Countries

**DOI:** 10.9745/GHSP-D-22-00472

**Published:** 2023-10-30

**Authors:** David Collins, Ulla Griffiths, Sarah Birse, Yohana Dukhan, Fadima Yaya Bocoum, Alfred Driwale, Humphries Nsona, Jerome Pfaffmann-Zambruni, Hannah Sarah F. Dini, Colin Gilmartin

**Affiliations:** a Boston University School of Public Health, Boston, MA, USA.; b UNICEF, New York, NY, USA.; c Management Sciences for Health, Medford, MA, USA.; d World Bank, Yaounde, Cameroon.; e Institut de Recherche en Sciences de la Sante, Bobo-Dioulasso, Burkina Faso.; f Independent consultant, Kampala, Uganda.; g Ministry of Health, Lilongwe, Malawi.; h UNICEF, Geneva, Switzerland.

## Abstract

**Background:**

Ensuring access to a package of integrated primary health care services is essential for achieving universal health coverage. In many countries, community health programs are necessary for primary health care service provision, but they are generally underfunded, and countries often lack the necessary evidence on costs and resource requirements. We conducted prospective cost analyses of community health programs in 6 countries in sub-Saharan Africa using the Community Health Planning and Costing Tool.

**Methods:**

The Community Health Planning and Costing Tool is a spreadsheet-based tool designed to cost key programmatic elements of community health services packages, including training, equipment, incentives, supervision, and management. In each country, stakeholders defined a package of community health services and corresponding standard treatment guidelines to estimate normative costs, which were applied to program scale-up targets. The data were entered into the tool, and cost models were prepared for different geographical and service utilization scenarios. The results were reviewed and validated with the governments, implementing partners, and expert panels. Additional scale-up scenarios were modeled, taking into account probable constraints to increasing community health service provision and potential funding limitations.

**Results:**

The services and scope of community health service packages varied by country, depending on contextual factors and determined health priorities. The package costs also varied significantly depending on the size and contents of the service package, the service delivery approach, the remuneration of the community health workers, and the cost of medicines and supplies.

**Conclusions:**

Community health programs and service packages are different in every country and change over time as they evolve. They should be routinely costed as an integral part of the planning and budgeting process and to ensure that sufficient resources are allocated for their effective and efficient implementation.

## Abstract (precis)

Authors of this article calculated the costs of implementing community health programs and compared the results across 6 sub-Saharan African countries, providing evidence for helping governments plan for sufficient resources for their effective implementation.

## Teaser

**Key Findings:**

- The Community Health Planning and Costing Tool is a dynamic analytic tool that was successfully applied in 6 countries in sub-Saharan Africa, providing evidence on costs and resource requirements for achieving universal access to integrated packages of primary health care provided by community health workers.
- Some of the programs covered in this study were designed before calculating the costs and faced challenges to achieve their goals. Therefore, it is crucial that costs be modeled and funding sources identified during the planning process so that the final program can be adequately resourced.

**Key Implications:**

- The scope and resource requirements of community health programs are different in every country, and therefore, each program must be costed individually when it is designed to facilitate resource mobilization and implementation.
- The Community Health Planning and Costing Tool can be used to cost community health programs and to model changes in services and unit costs on a regular basis.

## BACKGROUND

Community health services are essential in helping to achieve universal health coverage,^1^^–^^3^ as half of the world's population lacks access to essential health services,^4^ and countries face ongoing shortages of health workers.^5^ Community health services, especially those focused on integrated community case management (iCCM) for the treatment of childhood pneumonia, diarrhea, and malaria, can help to reduce high rates of mortality in many low- and middle-income countries.^6^ Community health services are a key component of primary health care (PHC) and are best defined as those provided by community health workers (CHWs) who live in the community that they serve.^7^ However, service packages can vary depending on several factors, including, but not limited to, community needs and epidemiology, available financial and human resources, and political will and influence. Community health service packages commonly comprise promotional services (e.g., promoting the use of latrines and hygiene), preventive services (e.g., organizing vaccination campaigns), basic curative services (e.g., diagnosis and treatment of malaria in children aged younger than 5 years), and others such as the distribution of family planning commodities and referrals to and from higher levels of care. Moreover, community-based approaches are becoming increasingly important for the prevention and treatment of noncommunicable diseases.^8^^,^^9^

PHC yields high returns on investment,^10^ with community health services expected to produce a 10:1 dividend when taking into account increased productivity from a healthier population, the avoidance of high costs of health crises, and the economic impact of increased employment.^11^ If well-utilized and provided efficiently, community health services should be less costly than those provided at facilities or mobile health clinics.^12^ Given the chronic shortage of skilled health workers, CHWs can support the overall PHC system by taking over appropriate tasks from facility-based providers—improving both efficiency and cost-effectiveness—while bringing services closer to the community. Moreover, to the degree that community health services substitute for those provided at facilities, the medicines and supplies required can be transferred from the facilities.

Community health services have been shown to promote access and utilization of services by reducing inequities relating to place of residence, gender, education, and socioeconomic status. Factors promoting greater equity of community health services include recruitment of women as CHWs, close proximity of services to households, preexisting social relationship with CHWs, free service delivery, targeting of poor households, strengthened referrals to facilities, and sensitization and mobilization of community members.^13^^–^^16^

Although community health services represent a cost-effective approach for the delivery of essential health services,^11^ they must be adequately resourced and supported to be of good quality and accessible.^17^ However, that has not often been the case, and they continue to face challenges of inadequate financing, lack of supplies and commodities, low compensation of CHWs, and inadequate training and supervision.^3^

Community health services represent a cost-effective approach for delivering essential health services, yet they continue to face challenges of inadequate financing, lack of supplies, low CHW compensation, and inadequate training and supervision.

To obtain the necessary financing, the required resources must be quantified and costed.^18^ Although there have been many studies of the resource and financing needs of vertical community programs, such as malaria or family planning,^15^^,^^19^ few have been conducted on comprehensive, integrated community health service packages, partly because no specific tool has been available. In 2016, UNICEF and Management Sciences for Health developed the Community Health Planning and Costing Tool (CHPCT). The tool was used to estimate the cost of meeting the need for community health services based on service utilization targets and normative costs in Angola, Burkina Faso, Madagascar, Malawi, Sierra Leone, and South Sudan. This article describes the approach used to calculate the costs of community health services in those countries and provides cost data that can be used as comparisons with the results of other studies.

## METHODS

### Costing Tool

The CHPCT was designed to cost packages of community health services from the provider perspective and to produce results to help assess performance, plan future services, and prepare investment cases for funding. It was based on the Integrated Community Case Management (iCCM) Costing and Financing Tool, which was developed by Management Sciences for Health in 2013 and was expanded to cover broader packages of community health services.^20^ Designed for use by health system managers and policymakers, the CHPCT is open source and spreadsheet based, allowing users to view and modify the structure and formulae. It calculates the normative costs of scaling up numbers of services using the resources needed to provide services with good quality, and these costs can be compared with the actual costs incurred to identify the additional cost of scaling up. Normative costing is important to identify the resources needed as opposed to historical costs that can be constrained by funding limitations, supply issues, or allocation problems.^21^

Users can calculate the costs of all elements of comprehensive community health service packages, including start-up, capital, training, and service delivery costs, as well as supervision and management costs at all levels of the health system. It also has a financing element that can be used to show program financing sources and gaps in current and future funding. The tool uses a bottom-up, ingredients-based method to calculate the normative costs.

The tool is split into 3 main sections: data entry, results, and intermediate calculations. Data entered in the tool are linked to the results, so a change in any data item will change the results. The tool and handbook (available in English and French) can be accessed via the available link.^22^

### Data Collection, Entry, and Reporting

**FIGURE 1:**
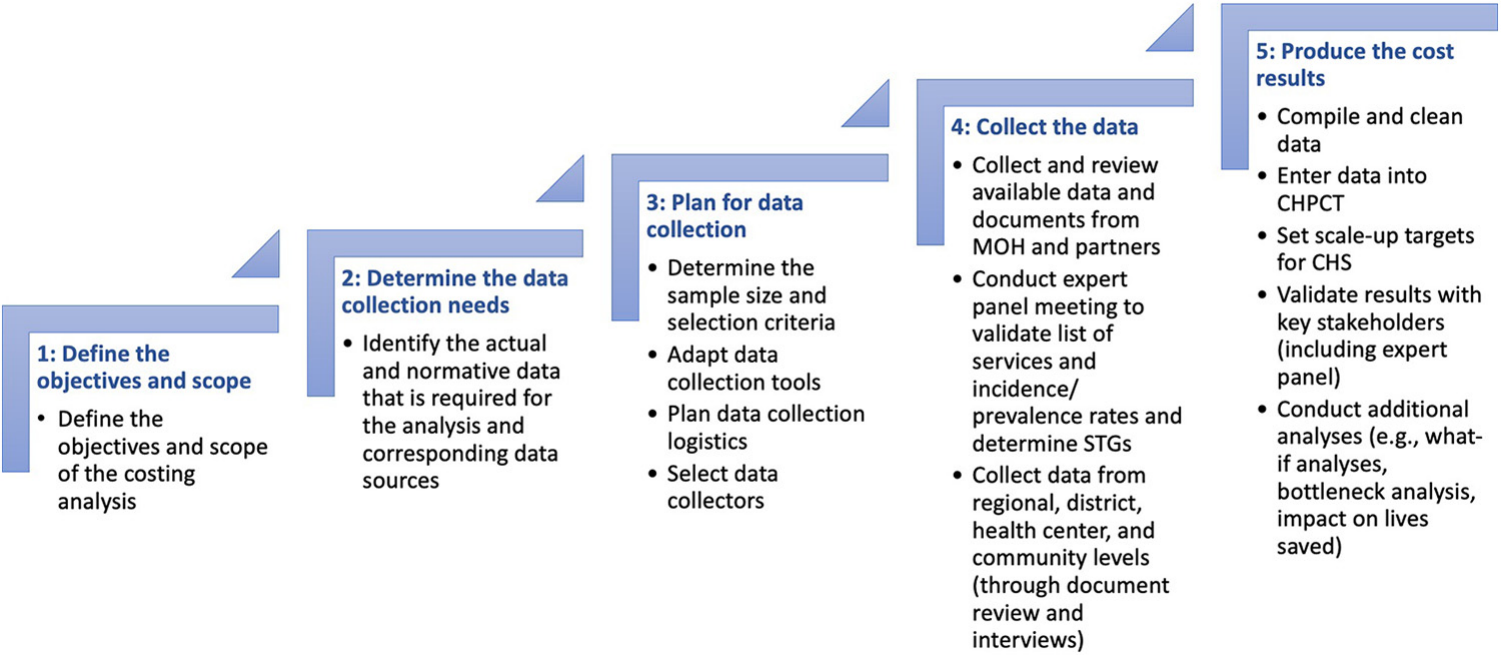
Community Health Service Costing Approach Abbreviations: CHPCT, Community Health Planning and Costing Tool; CHS, community health service; MOH, ministry of health; STG, standard treatment guideline.

The approach used for costing services and determining the corresponding financing needs involved 5 sequential steps (Figure 1). The required time for each country analysis depended on the size and complexity of the service package and the ease of data collection. Most of the studies were carried out by teams of 2 persons with 1 month in each country and an additional 4 to 6 weeks to finish the analysis, review final findings with the country government team, and write the report.

Step 1 involved determining the objectives and scope of each country's analysis with key stakeholders. Key decisions included, but were not limited to, determining the time horizon of the analysis, the explicit package of health interventions being assessed, and the geographical scope of the costing (i.e., whether its focus was at the national, provincial/regional, district, or subdistrict level). In all countries, the costing analysis was conducted from the program perspective (excluding patient and societal costs). Also, an expert panel was formed during this step.

**FIGURE 2:**
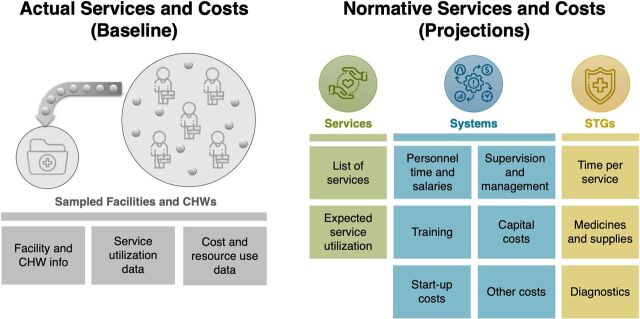
Data Collection Overview^23^ Abbreviation: CHW, community health worker, STG, standard treatment guideline.

Step 2 focused on determining the actual and normative data collection needs (Figure 2). This includes information on the interventions provided by CHWs; the estimated number, time, and costs of personnel supporting the program (e.g., CHWs, supervisors, and managers); expected costs of training and supervision; and costs of services based on standard treatment guidelines (STGs). Standard tools and checklists were used to facilitate the data collection process.

Steps 3 and 4 (data collection planning and execution) varied depending on the objectives and scope of each country analysis and whether extensive data collection at a sample of health facilities was required, as was the case in Malawi and Sierra Leone. In the 4 other countries, the majority of required data were collected from Ministries of Health and implementing partners at the national level. This included data on the number of existing (and anticipated) CHWs and corresponding health facilities for supervision and referral, service utilization data (disaggregated by service type), and expenditure data separated by resource type. Normative data on the explicit service package and expected annual utilization targets were based on the need for services, taking into account the catchment population, the incidence and prevalence rate of illnesses covered in the package, and expected utilization rates for preventive and promotional services, as well as the proportion of services likely to be provided by the CHWs, as opposed to other providers. Interviews with a sample of CHWs and their supervisors provided useful information on the organization of services, bottlenecks, and suggested improvements. These served as a reality check to the modeled findings. The interviews also provided insight on the travel and service delivery time that CHWs require for their activities. STGs were based on data from national documents and interviews as well as international guidelines. The role of the expert panel was important, especially for providing guidance on the annual utilization targets and the STGs.

Step 5 (produce the cost results) involved compiling and cleaning the data, followed by data entry into the CHPCT. It is important to note that while the actual utilization figures, the number of CHWS, and the actual salaries can be entered in the tool for the baseline year, the quantities and costs of equipment, training, supervision/management, other recurrent costs and start-up/capital costs are all normative. Medicines and supplies quantities are based on actual numbers of services, but normative prices are used. These proxies for actual costs were used for baseline years because it was not feasible in any of the countries to obtain actual expenditure figures.

Annual targets were adjusted to take into account current utilization levels (where available) and bottlenecks to scaling up. To calculate the normative cost for each service, the user entered the required CHW time and quantities of medicines and supplies into the STGs. The user also entered the numbers of CHWs and their remuneration, which was based on current levels or on proposed new levels, and the prices of medicines and supplies, which were based on current or projected government, market, or donor figures. Normative operational costs were also entered, and these were allocated across the services based on the normative time required by the CHW. These typically include items such as supervision and management costs, training costs, phone charging, bicycle repairs, and office running costs. Capital costs and start-up costs were entered separately from recurrent costs.

The reports produced by the tool showed the costs for each year by resource type, program, and individual service, permitting different types of analysis. They also showed the unit and total cost of each service (e.g., malaria diagnosis and treatment) broken down by resource type. The tool also reported the numbers of CHWs to be engaged and trained to replace those lost to attrition, average numbers of services per CHW, the total time spent on each service, and their productivity. It also produced a report showing the total quantity and cost of each type of medicine and supply used by the CHWs for the purpose of procurement planning. Several financing reports showed the sources of financing and financing gaps. When the first version of the tool was completed for a country or district, it was then used to develop a different model for each scenario by changing a few key assumptions. In the case of South Sudan and Burkina Faso, the costs produced by the tool were used to develop investment cases, with the addition of mortality and morbidity impact analyses using the Lives Saved Tool (LiST).

### Stakeholder Engagement and Expert Panel

The engagement of national stakeholders was an important part of the costing process, for example, for achieving consensus on the package of community health services, determining future program coverage targets (while identifying bottlenecks impeding its achievement), and informing policy discussions through what-if analyses. For example, in Madagascar, stakeholders defined a basic and expanded package of community health services, the latter representing a package that the Ministry of Health could offer in the future, depending on its resource envelope. They also used the tool to determine the cost implication of paying CHWs monthly stipends. In South Sudan, program coverage was reduced when the costing showed that there would not be enough funding to cover the whole country.

Expert panels played a critical role in defining the service package, validating or determining the STG for each service, and reviewing relevant data and results. This included reviewing incidence rates for diseases and CHW, supervisor, and manager time requirements, which are often overestimated by interviewees. In some countries, the panels were engaged throughout the process to provide guidance and feedback and to review findings. Panel composition differed across the countries, with members of different ministry of health departments that are involved in planning and managing community health services, managers and direct supervisors (e.g., health center nurses and peer supervisors) of CHWs who are closely involved in the provision of community health services, and, where possible, some senior CHWs.

## RESULTS

### Country Use Cases

Each country had different cost objectives in terms of the geographic focus of the analysis and modeled services and scale-up scenarios (Table 1). In Malawi,^23^ the existing community health service package was costed for 2 districts for 2015–2025 with different coverage options. In Sierra Leone,^24^ the new national service package was costed in 2 districts for 2015–2025, and the results were used to inform the National Community Health Strategy (2016–2020).^25^ In Madagascar,^26^ national-level costs were estimated for 2017–2026 for basic and expanded service package options, each with medium and high utilization levels. In South Sudan,^27^ the costing was done for a new national service package for various national and subnational scenarios for 2018–2028, and the results informed the national community health services strategy (*Boma Health Initiative*). In Burkina Faso,^28^ a new national community health services package was costed for the years 2018–2023, and different scale-up options were analyzed. In Angola,^29^ the costs were calculated for expanding the national *agentes de desenvolvimento sanitario e comunitario* (community development and sanitary agents) program to cover more service types for 2018–2028 with 3 scenarios—for all rural areas, for all urban areas, and for 1 province. In both Madagascar and South Sudan, the defined service packages were intended to harmonize fragmented community health programs that lacked a standardized approach to training, supportive supervision, and reporting, among other key programmatic elements.

**TABLE 1.** Overview of Country Use Cases of Community Health Service Packages

Country	Years Covered	Geographical Areas	Community Health Service Packages	Community Health Workers Accredited/Remunerated
Malawi	2015–2025	Ntcheu and Dedza Districts	Existing national service package	Yes/yes
Sierra Leone	2015–2025	Kono and Bombali Districts	New national service package	Yes/yes
Madagascar	2017–2026	National	Hypothetical basic and expanded service packages	Yes/no
South Sudan	2018–2028	National and various subnational scenarios (i.e., small, medium, and high coverage)	New national service package	Yes/yes
Burkina Faso	2018–2023	National	New national service package	Yes/yes
Angola	2018–2028	National rural, national urban, Malanje Province	New basic service package	Yes/yes

In Malawi and Sierra Leone, the actual numbers of community health services provided in the baseline year were available, and it was possible to calculate the gaps between actual and expected utilization and to set targets that were based on both sets of figures. However, in the other countries, actual numbers of community health services were not available, so desired utilization figures were used.

### Packages of Community Health Services

The scopes of work of the CHWs were based on the packages of community health services, which varied among the 6 countries (Table 2), ranging from 11 services in Angola to 43 in Malawi. In Malawi, the service package that was costed was the same as the one provided at the time, whereas in Angola, Madagascar, and Sierra Leone, the costing reflected an expansion of the existing package. In South Sudan and Burkina Faso, the package was a harmonized set of services to replace different packages currently provided in-country. Nascent community health programs (Angola and South Sudan) tended to have smaller service packages, mainly consisting of preventive and promotional services, while more mature programs (e.g., in Malawi) had larger service packages.

**TABLE 2.** Comparison of Packages Across Countries Showing Key Services^a^

	Malawi	Sierra Leone	Madagascar	South Sudan	Burkina Faso	Angola
Number of services costed	43	42	27	18	28	11
Service category						
Family planning	Yes	Yes	Yes	–	Yes	–
Antenatal and postnatal care	Yes	Yes	Yes	Yes	Yes	–
Pregnancy testing	–	–	Yes	–	–	–
Malaria treatment, children aged 5 years and younger	Yes	Yes	Yes	Yes	Yes	Yes
Diarrhea treatment, children aged 5 years and younger	Yes	Yes	Yes	Yes	Yes	Yes
Pneumonia treatment, children aged 5 years and younger	Yes	Yes	Yes	Yes	Yes	Yes
Malaria treatment, children aged 5 years and older	–	Yes	–	–	Yes	–
Immunizations	Yes	–	–	Yes	–	–
Support for TB control	Yes	–	–		Yes	Yes
HIV prevention and program support	Yes	–	–	Yes	Yes	–
Nutrition activities	Yes	Yes	Yes	Yes	Yes	
Routine household visits	–	Yes	Yes	–	–	Yes
Health promotion	Yes	Yes	Yes	Yes	Yes	Yes
Births and deaths reporting	–	Yes	–	–	–	–
Surveillance	–	Yes	Yes	Yes	Yes	–

a This list shows key differences across the countries and is structured differently from the groupings by program shown in later tables.

CHW's scopes of work were based on the packages of community health services, ranging from 11 services in Angola to 43 services in Malawi.

### Multi-Country Cost Results

To illustrate the results of using the tool, we selected 1 year of 1 scenario model for each country. Years without start-up or capital costs were selected except in the case of Burkina Faso. All costs are shown in US$, converted, where necessary, from local currency at the rates in use at the time of the studies.

Program utilization targets ranged from 6% in Madagascar to 81% in Angola, and the targeted number of services per capita ranged from 0.71 in Madagascar to 5.47 in Sierra Leone (Table 3). The average targeted number of services per hour per CHW ranged from 0.5 in Madagascar to 2.3 in Sierra Leone. The CHWs would be underutilized in 4 of the countries, with the lowest productivity rate (percentage of time worked over time available) being 22% in Madagascar. In Angola, the productivity rate would be 100% because the utilization rate was set to achieve that. Whereas in Sierra Leone, the rate would be 254%, which would mean that the CHWs would have to work for 50 hours per week rather than the policy of 20 hours per week (which led to a later request to model an increase in CHW remuneration).

**TABLE 3.** Community Health Service Program Targets and Costs,^a^ by Country Use Case

	Malawi Ntcheu District, 2019	Sierra Leone Kono District, 2019	Madagascar National Basic Medium, 2019	South Sudan 50%, 2019	Burkina Faso Medium, 2020	Angola Malanje, 2022
Target population	208,118	581,998	26,744,721	3,550,624	22,184,060	873,199
Number of service types in package	43	42	27	18	28	11
Average utilization across all services	30%	47%	6%	71%	15%	81%
Number of services	582,926	3,181,692	19,032,454	9,924,737	17,063,777	4,463,817
Average total services per capita	2.80	5.47	0.71	2.80	0.77	5.11
Promotive services per capita	0.00	1.42	0.02	0.28	0.04	1.87
Preventive services per capita	1.90	1.09	0.56	1.26	0.53	–
Curative services per capita	0.90	2.96	0.09	1.20	0.20	3.07
Surveillance, referral, reporting etc. services per capita	–	–	0.04	0.06	–	0.17
Number of CHWs	308	1,346	38,507	14,795	12,376	1,533
Population per CHW	676	432	695	240	1,793	569
CHW hours worked per week	30	20	20	20	15	40
Average services per CHW per week	36	45	10	13	27	56
Average services per hour	1.2	2.3	0.5	0.6	1.8	1.4
Average CHW productivity	32%	254%	22%	46%	59%	100%
Total recurrent cost, US$	1,189,050	2,357,677	6,568,562	15,305,472	27,324,404	10,593,839
Total capital cost, US$	–	–	–	–	5,227,404	–
Average recurrent cost per service, US$	2.04	0.74	0.35	1.54	1.60	2.37
Average recurrent cost per capita, US$	5.71	4.05	0.25	4.31	1.23	12.13
Average recurrent cost per CHW, US$	3,861	1,752	171	1,035	2,208	6,909
Average salary per CHW, US$	1,337	391	0	386	600	1,958

Abbreviation: CHW, community health worker.

a Each service has a different utilization target based on the population in need. These figures are the averages across all services.

The average recurrent cost per service ranged from US$0.35 in Madagascar to US$2.37 in Angola, and the average cost per capita ranged from US$0.25 in Madagascar to US$12.13 in Angola (Table 3 and Table 4). Capital costs were only planned in Burkina Faso, and these were excluded from the recurrent costs shown in the results. Thirty-five percent of the recurrent expenditure in the Burkina Faso model was not directly related to the delivery of services (e.g., insurance schemes) (Table 5). The highest volume services were reproductive health and family planning in Malawi and Madagascar; iCCM in Sierra Leone and Angola; maternal, newborn, and child health in South Sudan; and community mobilization and health promotion in Burkina Faso (Table 4 and Table 6). The highest cost services were reproductive health and family planning in Malawi; maternal, newborn, and child health in South Sudan; and iCCM in Sierra Leone, Madagascar, Burkina Faso, and Angola (Table 4 and Table 7). The cost of individual services also varied across the countries. For example, the cost of diagnosis and treatment of 1 case of pneumonia for a child aged younger than 5 years in 2019 would be US$0.78 in Madagascar and US$6.54 in Malawi, with the differences due to cost of CHW time and remuneration, dosages and prices of medicine, and in operational costs. The main cost drivers in most of the countries were CHW salaries and medicines and supplies, while supervision and training costs were significant in some countries (Table 5). In Madagascar, the picture was different because the CHWs did not receive any remuneration.

**TABLE 4.** Service Utilization and Cost per Capita,^a^ by Country Use Case

	Malawi Ntcheu District, 2019	Sierra Leone Kono District, 2019	Madagascar National Basic Medium, 2019	South Sudan 50%, 2019	Burkina Faso Medium, 2020	Angola Malanje, 2022
Reproductive health/family planning	1.12 ($2.33)	0.22 ($0.16)	0.39 ($0.06)	0	0.00 ($0.02)	0
Maternal, newborn, and child health	0.11 ($0.15)	0.44 ($0.07)	0.01 ($0.03)	1.50 ($2.64)	0	0
Integrated community case management^b^	1.05 ($1.87)	3.01 ($1.83)	0.14 ($0.13)	0	0.15 ($0.45)	2.69 ($7.45)
Malaria (aged 5 years and older)	0.00 ($0.00)	1.48 ($0.75)	0	0	0.04 ($0.13)	0
TB and HIV/AIDS	0.19 ($0.31)	0	0	0	0.00 ($0.00)	0.37 ($0.11)
Nutrition	0.02 ($0.01)	0.06 ($0.02)	0.13 ($0.00)	0	0	0
Community mobilization and promotion	0.00 ($0.00)	0.02 ($0.15)	0	0	0.50 ($0.27)	1.87 ($3.80)
Immunization	0.28 ($1.03)	0	0	0	0	0
Disease prevention and control	0	0.02 ($0.02)	0	0	0	0
Control of common communicable diseases	0	0	0	1.23 ($1.58)	0	0
Other	0	0.17 ($1.05)	0.01 ($0.03)	0.05 ($0.09)	0.06 ($0.36)	0.17 ($0.28)
Total utilization per capita	2.80	5.47	0.71	2.80	0.77	5.11
Total recurrent cost per capita	$5.71	$4.05	$0.25	$4.31	$1.23	$12.13

a Currency in US$.

b Malaria diagnosis and treatment for children aged younger than 5 years is included in integrated community case management.

**TABLE 5.** Total Cost by Resource Type and Percentages, by Country Use Case

	Malawi Ntcheu District, US$ (%)	Sierra Leone Kono District, US$ (%)	Madagascar National Basic Medium, US$ (%)	South Sudan 50%, US$ (%)	Burkina Faso Medium, US$ (%)	Angola Malanje, US$ (%)
	2019	2019	2019	2019	2020	2022
Community heath worker salaries	411,840 (35%)	525,709 (22%)	8 (0%)	5,716,788 (37%)	7,427,150 (23%)	3,003,047 (28%)
Equipment	7,578 (1%)	308,010 (13%)	1,054,768 (16%)	373,161 (2%)	184,497 (1%)	2,894,920 (27%)
Medicines, supplies, and commodities	463,109 (39%)	798,087 (34%)	1,950,297 (30%)	2,963,984 (19%)	1,657,888 (5%)	788,173 (7%)
Supervision	71,198 (6%)	69,496 (3%)	1,791,938 (27%)	1363660 (9%)	6,480,517 (20%)	512,134 (5%)
Training	150,479 (13%)	173,469 (7%)	1,711,634 (26%)	4,847,638 (32%)	116,104 (0%)	3,254,353 (31%)
Management	84,842 (7%)	455,046 (19%)	59,913 (1%)	40,240 (0%)	194,209 (1%)	120,809 (1%)
Other program costs	0 (0%)	27,856 (1%)	–	0 (0%)	11,264,036 (35%)	20,400 (0%)
Total	1,189,050	2,357,677	6,568,562	15,305,472	27,324,404	10,593,839

**TABLE 6.** Health Service Utilization Numbers and Percentages, by Country Use Case

	Malawi Ntcheu District	Sierra Leone Kono District	Madagascar National Basic Medium	South Sudan 50%	Burkina Faso Medium	Angola Malanje
	2019	2019	2019	2019	2020	2022
Reproductive health/family planning	235,107 (40%)	131,294 (4%)	10,682,866 (56%)	–	152,891 (1%)	–
Maternal, newborn, and child health	23,590 (4%)	261,094 (8%)	448,176 (2%)	5,346,409 (54%)	–	–
Integrated community case management	219,781 (38%)	1,753,409 (55%)	3,817,090 (20%)	–	3,371,479 (20%)	2,355,352 (53%)
Malaria (aged 5 years and older)	123 (0%)	864,912 (27%)	–	–	962,515 (6%)	–
TB and HIV/AIDS	41,121 (7%)	–	–	–	2,411 (0%)	327,135 (7%)
Nutrition	4,401 (1%)	38,425 (1%)	3,732,572 (20%)	–		–
Community mobilization and promotion	123 (0%)	16,601 (1%)	–	–	11,102,526 (65%)	1,632,886 (37%)
Immunization	58,679 (10%)	–	–	–	–	–
Disease prevention and control	–	12,957 (0%)	–	–	–	–
Control of common communicable diseases	–	–	–	4,370,069 (44%)	–	–
Other	–	102,951 (3%)	351,750 (2%)	208,260 (2%)	1,471,955 (9%)	148,444 (3%)
Total	582,926	3,181,692	19,032,454	9,924,737	17,063,777	4,463,817

**TABLE 7.** Total Cost by Health Program and Percentages, by Country Use Case

	Malawi Ntcheu District, US$ (%)	Sierra Leone Kono District, US$ (%)	Madagascar National Basic Medium, US$ (%)	South Sudan 50%, US$ (%)	Burkina Faso Medium, US$ (%)	Angola Malanje, US$ (%)
	2019	2019	2019	2019	2020	2022
Reproductive health/family planning	484,710 (41%)	93,693 (4%)	1,528,702 (23%)	–	376,124 (1%)	–
Maternal, newborn, and child health	32,043 (3%)	41,558 (2%)	934,713 (14%)	9,379,416 (61%)		–
Integrated community case management	388,943 (33%)	1,065,941 (45%)	3,405,525 (52%)	–	9,894,209 (36%)	6,940,588 (66%)
Malaria (aged 5 years and older)	227 (0%)	436,986 (19%)	–	–	2,889,896 (11%)	–
TB and HIV/AIDS	65,512 (6%)	–		–	4,122 (0%)	9,4176 (1%)
Nutrition	2,643 (0)%	10,019 (0%)	7,908 (0%)	–	–	–
Community mobilization and promotion	117 (0%)	84,647 (4%)	1,725 (0%)	–	6,074,803 (22%)	3,314,877 (31%)
Immunization	214,852 (18%)	–		–	–	–
Disease prevention and control	–	11,864 (1%)		–	–	–
Control of common communicable diseases	–	–	–	5,614,018 (37%)	–	–
Other	–	612,965 (26%)	689,986 (11%)	312,036 (2%)	8,085,250 (30%)	244,197 (2%)
Total costs	1,189,050	2,357,677	6,568,562	15,305,471	27,324,404	10,593,839

### Scenario Modeling

Sensitivity analyses were not conducted systematically as part of the country studies. However, models were developed for different scenarios to take into account possible constraints to scaling up. Such scenarios were easily done using the CHPCT, as all programmatic variables and assumptions are linked to the total cost. In Malawi and Sierra Leone, where the tool was first piloted, different scenarios were not developed, although, in Sierra Leone, the cost of increasing remuneration for CHWs was modeled at the request of the Ministry of Health and Sanitation.

In Madagascar, scenarios were developed for different service package models for different coverage levels. The South Sudan study was based on 3 scenarios—low, medium, and high coverage—and 4 new lower-cost scenarios were developed later based on reduced geographical coverage. In Burkina Faso, models were developed for low, medium, and high coverage for 3 regions, each with slightly different service packages. In Angola, 3 models were developed: 1 for the rural population, 1 for the urban population, and 1 for 1 province with the highest need for iCCM services.

## DISCUSSION

This article aimed to describe the approach used to calculate the costs of community health services and to provide cost data that can be used as comparisons with the results of other studies. In this section, we discuss the lessons learned from carrying out the costing and comparing results across countries, and we reflect on the interpretation of costs for the broader health system.

The approach used to calculate the costs produced sufficient detailed information for strategic and operational planning and financing, as well as for comparing costs across studies. The usefulness of the results of the costing studies was greater in countries where national stakeholders were engaged in the process and where the costing was part of the planning process. Having a respected and knowledgeable expert panel was important for validating or developing reasonable estimates for CHW travel and service delivery time, supervision and management time, and incidence and prevalence rates. The expert panel was also helpful in setting service delivery targets that take into account the supply- and demand-side bottlenecks, as well as supporting and explaining the results to stakeholders.

Meaningful comparisons of costs from different countries are difficult because the types and quantities of services and the quantities and costs of required resources can all vary considerably. This analysis shows that there is no standard service package or delivery method for community health programs because they have to address the differing health needs of their people and be based on the local situation, such as population density, disease burden, geographical access, security issues, and cultural habits. Variations in service delivery mechanisms and resource prices mean that direct and indirect costs can differ considerably. Capital and start-up costs that are incurred in some years affect the total costs as well as the average cost per capita and per service and the cost of individual services. Recurrent costs can vary across countries due to differences in CHW remuneration, normative estimates of service delivery times, and differences in medicines dosages and prices. They can also vary over years due to factors such as increases in resource prices, greater program efficiency, and economies of scale. When trying to compare costs across countries or over time, it is essential to take into account all of the major factors that affect them. The tables included in this article provide examples of these factors. An additional report, which is not shown, provides the unit and total cost for each service, including a breakdown of the unit cost by input type. Additional information that would be useful would be the standard travel time for each service and average travel time for all services, which are not provided in the tool reports but could be with a simple modification.

Only 1 published article on the costs of integrated packages of community health services in low- and middle-income countries could be identified, which was conducted in Mali in 2016–2019 by Saint-Firmin et al.^30^ This article contained comprehensive data and analysis, but it is not clear if it is sufficient for a meaningful comparison with the results from the 6 countries as previously described. The study used the CHPCT to calculate the cost of a package of 23 curative, preventive, and promotional interventions. It found that the program spent US$13.01 million (US$10.50 per service) but could have achieved the same service volume with US$8.36 million (US$6.80 per service) if STGs had been followed. A 2015 literature review by Vaughan et al. found 32 studies of the cost of community health programs.^15^ However, all of these only covered individual services or programs, and none of these covered comprehensive packages of community health services. A cross-country study of the costs of iCCM services in 6 countries published in 2014 only covered those services.^19^ A literature review of CHW programs in humanitarian settings published in 2020 did not include any information on costs.^31^

The 6 country studies identified some key areas of design that affect a program's cost and effectiveness: the location where services are provided, the use of available time of the CHWs, and the structure of supervision. If CHWs have to cover a large area and provide services from their homes, clients may delay seeking help or may incur opportunity costs and/or out-of-pocket costs. However, if CHWs travel to clients' homes, they may incur out-of-pocket costs and will have less time for other services. In the first case, the program cost per service will be lower and the client cost higher, while the opposite will be true in the second case. If CHWs make routine visits to every household, with priority to those with vulnerable members, it may be possible for them to provide several services in 1 visit, which could be cost-effective. Regular high-quality supervision is important to ensure the quality and continuity of service provision and can serve as a motivating factor for CHWs.^32^ However, it can also be costly, especially when visits are frequent and require transportation. Therefore, it is important to develop cost-effective supervision methods (e.g., by combining facility supervisor visits to communities with other activities such as providing some curative services and providing health promotion talks). Some countries use a combination of visits from supervisors to the communities, peer supervision models, and group CHW meetings at the facility.

The country studies identified areas of program design that affect a program's cost and effectiveness: the location where services are provided, the use of available time of the CHWs, and the structure of supervision.

A community health program is part of a network of PHC services, and it is important to note that the full cost of the program may not reflect an additional cost to the network or to the community. A community health program can reduce the burden on the health center for services that can be carried out by CHWs and through preventive interventions. This can either save health center resources or allow the resources to be used more cost-effectively. It can also lower health center costs per service by improving efficiency, for example, by supporting vaccination campaigns and by following up on clients treated at the health center. Although CHWs can increase the health center burden for some services by identifying and referring ill persons they cannot treat, this referral may benefit the clients by increasing the likelihood that they will be treated in good time and/or reducing costly visits to the hospital or to private providers. It is also important to note that some of the costs of the program may already be covered, such as the salaries of managers and supervisors. In such cases, the only additional management and supervision costs are those related to specific activities that would not be undertaken if the program did not exist, such as supervision travel specifically related to the program (not part of general supervision). Also, if the CHWs are using medicines and supplies to treat clients who would otherwise be treated at a facility, those costs are not additional to the health system. If a CHW has fixed remuneration and spare time, the marginal additional cost of treating a sick child is the cost of the medicine, and if the child would otherwise have been treated at a facility and the medicines are transferred to the CHW, the marginal additional cost may be zero. There were insufficient resources to include any of these factors in the 6 country studies presented in this article, but they merit research in the future.

### Limitations

The limitations with the costing results shown in the article relate mainly to the difficulties in obtaining accurate, timely, good-quality data, which are common for this type of costing study. Catchment populations that were used to calculate actual per capita utilization rates and costs were based on political boundaries, which may not have always reflected the actual populations that should be covered by CHWs. Aggregated data on the actual utilization of CHW services were not usually available for baseline years because they were not shown separately in the national health information systems or because services were mainly provided by nongovernmental organizations. Actual expenditure figures for some inputs were also not available because they were included with other expenditures at the district level or because they were incurred by different nongovernmental organizations.

Utilization targets for iCCM services were based on incidence rates for malaria, pneumonia, and diarrhea, which were not available in all countries, especially at subnational levels. There were often no accurate estimates of care-seeking behavior for some services provided by the CHWs, such as the proportion of families that would take their children with suspected malaria to a CHW versus self-treatment or care-seeking at a public or private facility. National or local targets for promotional and preventive services were also not always available. It was not always possible to estimate the impact of bottleneck constraints. Estimates of travel and service delivery times time were obtained through CHW interviews but had wide variations and a tendency for overestimation. These had to be adjusted based on reviews by supervisors and the expert panel.

Due to these challenges, the coverage targets and related costs are best regarded as indicative. The different scenarios showed the results of changes in packages, coverage targets, and CHW remuneration, and these were made clear to the stakeholders so that they appreciated the degree to which results were based on assumptions.

Care must be taken with the interpretation of results because the tool uses the targeted numbers of services as the basis for calculating the cost of medicines and supplies, but the costs related to the CHWs, supervisors, and managers are not automatically related to the numbers of services. The user can enter the target number of CHWs per population, per community, or manually, but not based on need. This can result in underutilization or overutilization of CHWs in a year, so it is important to look at the CHW productivity figures when reviewing and comparing results. Low productivity, as in the case of Ntcheu District, Malawi, means that the costs were higher than they should have been. However, the excessive demands on CHW time in Kono District, Sierra Leone, means that they would have to work for much longer than their official hours and they would probably have to be paid more (or more CHWs would have to be engaged), so costs were lower than they should be. The effects of CHW productivity on costs are magnified because quantities of CHW equipment and training are driven by the numbers of CHWs, as are the number of supervisors and their equipment.

Care must also be taken with the interpretation of results related to the inclusion of capital and start-up costs. Capital costs are recorded when they are incurred and are not depreciated. Start-up costs generally relate to the cost of providing initial training to CHWs who are added when a program is geographically expanded. Capital and start-up costs are not normally incurred every year, so total costs can be higher in some years than in others. Although these costs are reported separately in the tool, they are included in all of the detailed costs (e.g., by service, by program, and by input type) and are, therefore, included in the average cost per service, per capita, and per CHW. Therefore, these figures must be removed from the calculations if it is desired to only show the recurrent costs.

## CONCLUSIONS

Recognizing the important role of community health programs in achieving universal health coverage, it is essential that they are accessible to persons in need and of high quality. To achieve these outcomes, programs must be feasible, affordable, efficient, effective, equitable, and sustainable. These studies show that community health service packages and delivery and support mechanisms can vary greatly, as do the costs of developing and maintaining them. As a result, there are no standard costs for community health service packages, and each must be costed separately. In many cases, programs are designed before having an awareness of the cost and then struggle to achieve their goals. Therefore, it is crucial that costs be modeled and funding sources identified during the planning process so that the final program can be adequately resourced. Modeling the costs allows for initial proposals to be ambitious and to be scaled back as needed when funding commitments become clear. A dynamic tool and simple approach allow for easy and quick changes in assumptions that can facilitate the compilation and comparison of results across countries and over time.

The costing approach and tool used for these studies proved feasible for this type and depth of modeling and analysis. The key elements of the studies that helped make it feasible were the use of normative unit costs, the use of only a small number of visits to facilities for additional data collection and validation, and the use of an expert panel to guide the studies and provide missing data.

In most of the countries, the results of the costing studies have been useful to the governments and partners in helping to prioritize and plan their community health programs. In South Sudan, as a result of the initial cost analysis, which indicated the high cost of covering the whole country, the government decided to focus on the high-priority rural areas.

Dynamic, accessible tools like the CHPCT are necessary to produce such models in a timely fashion and can be updated over time to reflect better data and changes in assumptions, such as input prices and incidence rates.

## Notes

**Peer Reviewed**

**First published online:** October 6, 2023.
